# Factors associated with glycemic control in patients with T2DM: evidence from a cross-sectional study in China

**DOI:** 10.1186/s12902-024-01605-5

**Published:** 2024-06-03

**Authors:** Wenting Luo, Jiayu Zhang, Yanxing Luo, Qiuwan Wu, Longfei Chen, Changqin Liu, Minqiang Lin

**Affiliations:** 1 Department of Scientific Research, School of Medicine, the First Affiliated Hospital of Xiamen University, Xiamen University, No.24, Bailu Road, Siming District, Xiamen, 361003 Fujian China; 2 Department of Intensive Care Medicine, School of Medicine, the First Affiliated Hospital of Xiamen University, Xiamen University, Xiamen, 361003 China; 3 School of Public Health, Fujian Medical University, Fuzhou, 350122 China; 4 Department of Endocrinology and Diabetes, School of Medicine, the First Affiliated Hospital of Xiamen University, Xiamen University, Xiamen, 361003 China; 5 Institute of Clinical Medical Research, School of Medicine, the First Affiliated Hospital of Xiamen University, Xiamen University, Xiamen, 361003 China

**Keywords:** Diabetes, Patient management, Glucose control, Influencing factors

## Abstract

**Objective:**

This study aimed to analyze the factors influencing glycemic control in patients with type 2 diabetes mellitus (T2DM).

**Methods:**

Baseline data, encompassing basic information, lifestyle habits, and treatment of 305 T2DM patients from March 2021 to January 2023, were collected and analyzed using SPSS 26.0 software.

**Results:**

Univariate and multivariate logistic regression analyses identified insulin therapy (OR = 2.233; 95%Cl = 1.013–4.520; *P* = 0.026) and regular clinic visits (OR = 0.567; 95%Cl = 0.330–0.973; *P* = 0.040) as independent factors influencing glycemic control. No observed interactions between the two variables were noted.

**Conclusion:**

History of insulin therapy and regular clinic visits were significantly and independently associated with glycated hemoglobin control in T2DM patients. Tailored interventions based on individual circumstances are recommended to optimize glycemic control.

## Introduction

Diabetes mellitus, characterized by chronic hyperglycemia due to insulin secretion defects, poses significant health and economic burdens [1]. Type 2 Diabetes Mellitus (T2DM) accounts for over 90% of diabetes cases and can lead to severe complications if not adequately controlled [2]. Continuous glucose monitoring aids in identifying patients’ conditions, allowing timely adjustments to treatment decisions [3]. Poor glycemic control has been associated with various factors, necessitating a comprehensive understanding of influencing factors for effective management. According to Al-Qerem et al. [3], a group of 287 participants from Amman, reported that the prevalence of inadequate glycemic control was 58%. In a cross-sectional study by Betelhem Demeke Habteyohans et al. [4], glycemic control was poor in two-thirds of the subjects. Plenty of studies have shown that various factors are closely associated with inadequate glycemic management.

There is a correlation between specific glycated hemoglobin and blood glucose concentration, which allows patients to gauge their glycated hemoglobin levels through self-testing of blood glucose, thus aiding in self-management and treatment. However, the results of previous cross-sectional studies were inconsistent. Also, most studies had limited sample size in T2DM patients. In addition, the prevalence of diabetes in China has increased rapidly in recent years [5]. This study aimed to investigate the factors that may affect Chinese patients’ glycohemoglobin control and to provide an accurate starting point for the management of patients with T2DM.

## Methods

### Participants

305 T2DM patients were recruited from the First Affiliated Hospital of Xiamen University, China, between March 2021 and January 2023. Informed consent was obtained, and the study was approved by the ethics committee of the First Affiliated Hospital of Xiamen University.

### Survey methods

The criterion for determining the achievement of glucose control was established based on the Chinese Guidelines for the Prevention and Control of T2DM with the following [6]. This involved simultaneous testing of fasting blood glucose, 2 h blood glucose after an oral glucose tolerance test (OGTT), and glycated hemoglobin, with a diagnostic threshold set at a glycated hemoglobin value of > 7.0%. Patients with type 1 diabetes mellitus, gestational diabetes mellitus, and other non-T2DM conditions were excluded from the analysis. Inclusion criteria required adherence to clinical diagnostic criteria for T2DM, while exclusion criteria encompassed (1) severe organic condition and (2) refusal to complete questionnaires and sign informed consent forms.

Following the specified criteria, patients were categorized based on their glycated hemoglobin levels. Those with values exceeding 7.0% were considered into the substandard control group, whereas those with values below 7.0% were assigned to the standard control group. Each subject received a face-to-face interview to collect sociodemographic data including education level, economic status, lifestyle habits and so on, present and previous health history, and medication utilization.

### Statistical analysis

All data were processed and analyzed utilizing Microsoft Excel and SPSS 26.0 software. Categorical variables were presented as frequencies with percentages (%), while continuous variables were presented as standard deviations (SD). Baseline characteristics between groups were compared using the chi-square test. Single-factor and multi-factor analyses of the blood glucose control were performed by binary logistic regression analysis, and odds ratios (OR) were calculated. A significance threshold of *P* < 0.05 was applied to determine statistical significance.

To further assess interaction effects, the parameter estimates and covariance matrices of the logistic regression model were calculated. Summed interaction indexes, including the relative excess risk of interaction (RERI), the attributable proportion of interaction (AP), and the synergy index (SI), were computed using the Excel sheet prepared by Andersson et al. [12]. The presence of an additive interaction effect between the two factors was indicated if the 95% confidence intervals (CI) of RERI and AP did not include 0, and the 95% CI of SI did not include 1.

## Results

### Characteristics of participants stratified by glycated hemoglobin

A total of 305 patients were enrolled in this study, consisting of 193 male patients and 112 female patients. Among these participants, the average age was 47.0 years (SD = 12.4 years). Most of the patients come from an urban area in Xiamen, China. Within the substandard glycemic control group, there were 155 cases, with 65.8% (102/155) male and 34.2% (53/155) female patients. In the standard glycemic control group, there were 150 cases, comprising 60.7% (91/150) male and 39.3% (59/150) female patients. The comprehensive analysis of fundamental information (including gender, education level, personal economic status, and type of occupation) did not reveal statistically significant differences between the groups (Table 1).

**Table 1:** Baseline characteristics in patients with T2DM

Observation indicators	Glucose control substandard group (*n* = 155)	Blood glucose control standard group (*n* = 150)	X^2^	*P*
**Sex (n, %)**			*0.867*	*0.352*
Male	102(65.8)	91(60.7)		
Female	53(34.2)	59(39.3)		
**Illiterate**			*2.244*	*0.691*
Illiterate	5(3.4)	6(4.1)		
Elementary school	21(14.1)	21(14.2)		
Junior high school	42(28.2)	34(23)		
High School/College	60(40.3)	58(39.2)		
College or above	21(14.1)	29(19.6)		
**Personal economic status**			*1.557*	*0.212*
Self-independent	139(90.8)	140(94.6)		
Dependent	14(9.2)	8(5.4)		
**Occupation type**			*2.729*	*0.604*
Unemployed	16(10.5)	16(10.7)		
Student	3(2)	2(1.3)		
Employed	100(65.4)	105(70.5)		
Retired	28(18.3)	18(12.1)		
Other	6(3.9)	8(5.4)		
**Exercise Habit**			*2.340*	*0.126*
Yes	78(50.3)	88(59.1)		
No	77(49.7)	61(40.9)		
**Eating Habits**			*0.374*	*0.879*
Balanced Meat and Vegetable	93(62.4)	95(65.1)		
Meat-based	41(27.5)	39(26.7)		
Vegetarian	15(10.1)	12(8.2)		
**Salt-loving diet**			*1.399*	*0.237*
Yes	60(38.7)	48(32.2)		
No	95(61.3)	101(67.8)		
**Oily diet**			*0.299*	*0.585*
Yes	63(40.6)	56(37.6)		
No	92(59.4)	93(62.4)		
**Sugar habit**			*2.358*	*0.125*
Yes	17(11.0)	9(6.0)		
No	138(89.0)	140(94.0)		
**Smoking history**			*0.269*	*0.604*
Yes	43(28.9)	39(26.2)		
No	106(71.1)	110(73.8)		
**History of alcohol consumption**			*1.658*	*0.198*
Yes	47(31.5)	37(24.8)		
No	102(68.5)	112(75.2)		
**History of high blood pressure**			*0.562*	*0.453*
Yes	31(20.7)	25(17.2)		
No	119(79.3)	120(82.8)		
**Previous history of hyperlipidemia**			*3.679*	*0.055*
Yes	48(32.0)	32(22.1)		
No	102(68.0)	113(77.9)		
**History of gout**			*0.835*	*0.361*
Yes	5(3.3)	8(5.5)		
No	145(96.7)	137(94.5)		
**History of thyroid disease**			*0.003*	*0.957*
Yes	5(3.3)	5(3.4)		
No	145(96.7)	140(96.6)		
**History of drug allergy**			*0.187*	*0.665*
Yes	9(5.8)	7(4.7)		
No	146(94.2)	142(95.3)		
**Family history of diabetes**			*0.763*	*0.382*
Yes	153(98.7)	145(97.3)		
No	2(1.3)	4(2.7)		
**Used lifestyle interventions** ^**#**^			***4.195***	***0.041***
Yes	48(31.0)	63(42.3)		
No	107(69.0)	86(57.7)		
**Oral hypoglycemic drugs**			*0.778*	*0.378*
Yes	103(66.5)	106(71.1)		
No	52(33.5)	43(28.9)		
**Insulin therapy**			*0.899*	*0.343*
Yes	36(23.2)	28(18.8)		
No	119(76.8)	121(81.2)		
**Taking oral medications**			*0.843*	*0.358*
Yes	93(60.0)	97(65.1)		
No	62(40.0)	52(34.9)		
**Taking insulin injections** ^**#**^			***5.397***	***0.020***
Yes	33(21.3)	17(11.4)		
No	122(78.7)	132(88.6)		
**Regular medical appointments** ^**#**^			***8.226***	***0.004***
Yes	68(45.0)	90(61.6)		
No	83(55.0)	56(38.4)		
**Regular use of medication**			*0.047*	*0.829*
Yes	85(56.3)	84(57.5)		
No	66(43.7)	62(42.5)		
**Self-testing of blood glucose** ^**#**^			***4.686***	***0.030***
Yes	64(41.3)	80(53.7)		
No	91(58.7)	69(46.3)		
**Family History**			*0.228*	*0.633*
Yes	100(64.5)	100(67.1)		
No	55(35.5)	49(32.9)		

The variables are assigned the following values. Sex: female = 0, male = 1; Literacy: illiterate = 0; elementary school = 1, middle school = 2; high school/junior college = 3; college or above = 4; Personal economic status: dependent = 0; self-independent = 1; Occupation type: unemployed = 0; student = 1; employed = 2; retired = 3; other = 4; Exercise habits: none = 0, yes = 1; Dietary habits: meat/vegetable balanced = 0; meat-based = 1; veg. Dietary habits: meat-vegetable balance = 0; meat-based diet = 1; vegetarian diet = 0; salt-addicted diet: no = 0, yes = 1; oil-addicted diet: no = 0, yes = 1; sugar-addicted diet: no = 0, yes = 1; history of smoking: no = 0, yes = 1; history of drinking: no = 0, yes = 1; history of hypertension: no = 0, yes = 1; history of high blood pressure: no = 0, yes = 1; history of gout: no = 0, yes = 1; history of thyroid disease: no = 0, yes = 1; history of drug allergy: no = 0, yes = 1; history of drug allergies: no = 0, yes = 1; family history of diabetes mellitus; no = 0, yes = 1; what treatment modality was used: no = 0, yes = 1; what treatment modality was used lifestyle intervention: no = 0, yes = 1; oral hypoglycemic agents: no = 0, yes = 1; what treatment modality was used insulin: no = 0, yes = 1; whether currently taking oral medications: no = 0, yes = 1; Whether currently injecting insulin: no = 0, yes = 1; past regular medical visits: no = 0, yes = 1; regular medication use: no = 0, yes = 1; self-testing of blood glucose: no = 0, yes = 1; family history: no = 0, yes = 1. Statistical significance is indicated by “#”

### Associations between glycemic control and risk factors

The chi-square analysis revealed noteworthy differences in therapeutic lifestyle interventions, insulin therapy, previous clinic visits, and self-monitoring of blood glucose between the substandard and the standard glycemic control groups.

Results from univariable logistic regression analysis exhibited statistical significance for glycemic control about various factors. Specifically, the utilization of any treatment lifestyle intervention (OR = 0.612, *P* = 0.041), current insulin injections (OR = 2.1, *P* = 0.022), regular medical appointments (OR = 0.51, *P* = 0.004), and self-testing of blood glucose (OR = 0.607, *P* = 0.031) all played a significant role. Adherence to specific treatment modalities, regular medical visits, and self-testing of blood glucose were associated with favorable glycemic control, while current insulin injections were found to be for control (*P* < 0.05) (Table 2).

**Table 2:** Univariable logistic regression analysis

Observation indicators	Glucose control substandard group (*n* = 155 )				Blood glucose control standard group (*n* = 150)				*P*		*OR*		*OR95%CI*		
**Used lifestyle interventions** ^#^															
Yes		107(69.0)			86(57.7)						*1*				
No		**48(31.0)**			**63(42.3)**				***0.041***		***0.612***		***0.382***		***0.98***
**Taking insulin injections** ^#^															
Yes		122(78.7)			132(88.6)						*1*				
No		**33(21.3)**			**17(11.4)**				***0.022***		***2.1***		***1.113***		***3.962***
**Regular medical appointments** ^#^															
Yes		83(55.0)			56(38.4)						*1*				
No		**68(45.0)**			**90(61.6)**				***0.004***		***0.51***		***0.321***		***0.81***
**Self-testing of blood glucose** ^#^															
Yes		91(58.7)			69(46.3)						*1*				
No		**64(41.3)**			**80(53.7)**				***0.031***		***0.607***		***0.385***		***0.955***

Statistical significance is indicated by “#”

In-depth multifactorial logistic regression analysis demonstrated the statistically significant impact of current insulin therapy and regular clinic visits on glycemic control (*P* < 0.05). This significance persisted even after adjusting for confounding factors, such as gender, education level, personal economic status, type of occupation, and others. The effects of current insulin therapy and regular clinic visits on glycemic control remained statistically significant (*P* < 0.05) (Table 3).

**Table 3:** Results of multifactorial logistic regression analysis

Variant	Before adjustment			After adjustment*	
	OR95%CI	*P*		OR95%CI	*P*
**Used lifestyle interventions**					
Yes	*1.000*			*1.000*	
No	*0.729(0.443 ~ 1.202)*	*0.216*		*0.729(0.428 ~ 1.243)*	*0.246*
**Taking insulin injections** ^#^					
Yes	*1.000*			*1.000*	
No	*2.444(1.253 ~ 4.766)*	*0.009*		*2.233(1.103 ~ 4.520)*	*0.026*
**Regular medical appointments** ^#^					
Yes	*1.000*			*1.000*	
No	*0.579(0.345 ~ 0.970)*	*0.038*		*0.567(0.330 ~ 0.973)*	*0.040*
**Self-testing of blood glucose**					
Yes	*1.000*			*1.000*	
No	*0.664(0.401 ~ 1.099)*	*0.111*		*0.590(0.346 ~ 1.006)*	*0.111*

* Adjustment for gender, literacy, personal economic status, type of occupation. Statistical significance is indicated by "#".

### Interaction analysis

Multiplicative interaction analysis was undertaken to evaluate the interactive impact of current insulin injections and regular clinic visits on glycemic control in diabetic patients. The logistic regression model included independent variables such as current insulin therapy, regular clinic visits, and the interaction term (current insulin therapy × regular clinic visits). Both pre-adjusted and adjusted models were formulated, and the outcomes revealed an absence of multiplicative interaction between the two influences, both before and after adjusting for confounding variables (Table 4).

**Table 4:** Results of multiplicative interaction analysis

Variant	Before adjustment			After adjustment*	
	OR95%CI	*P*		OR95%CI	*P*
Taking insulin injections	*2.414(0.744 ~ 7.831)*	*0.142*		*2.314(0.683 ~ 7.838)*	*0.178*
Regular medical appointments	*0.473(0.285 ~ 0.785)*	*0.004*		*0.456(0.267 ~ 0.777)*	*0.004*
Taking insulin injections×Regular medical appointments	*0.951(0.230 ~ 3.928)*	*0.945*		*0.894(0.206 ~ 3.872)*	*0.881*

* Adjustment for gender, literacy, personal economic status, type of occupation

**Fig. 1:**
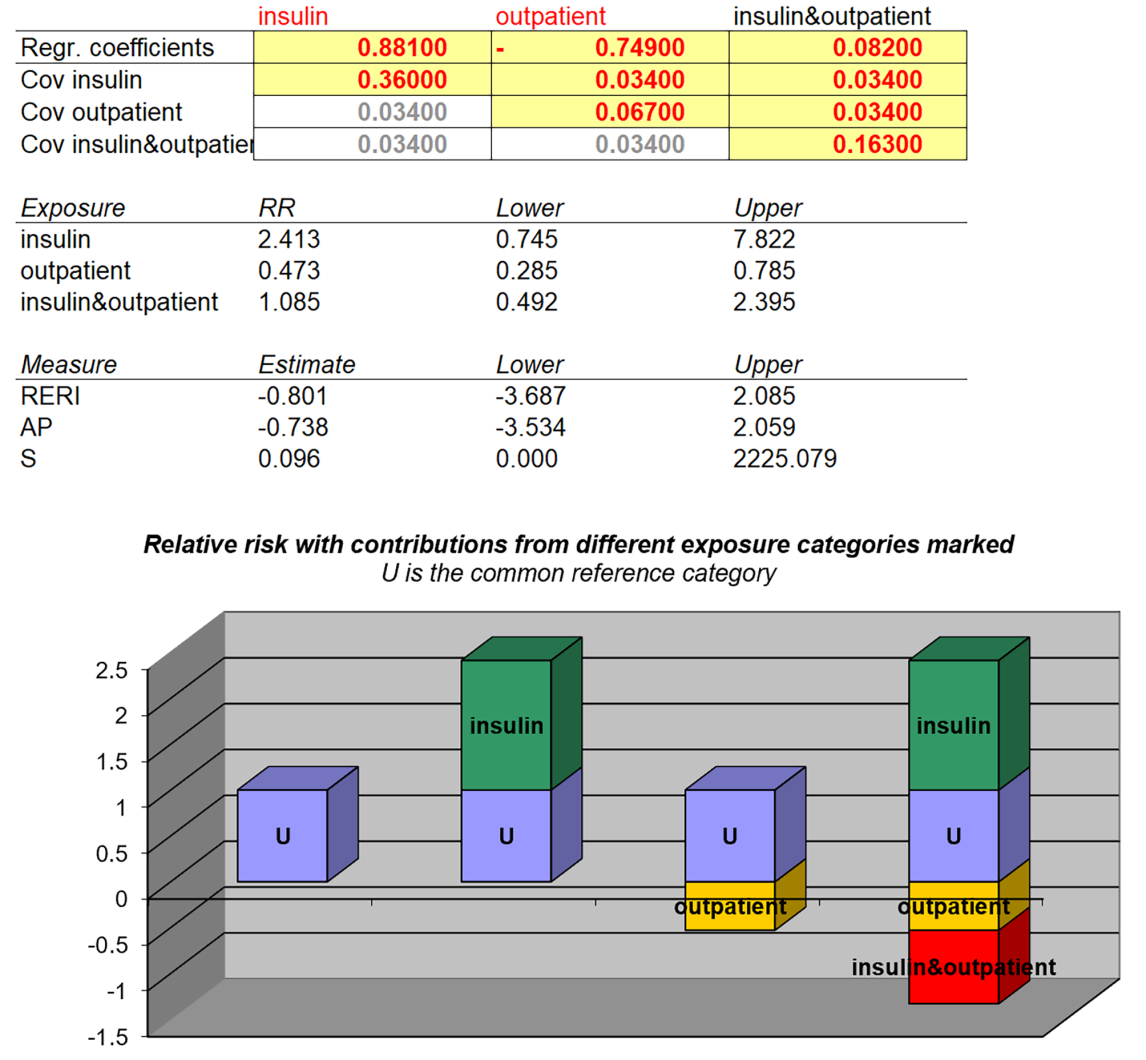
Association between exposure factors and glycemic control in patients with T2DM

To further explore the additive interaction between current insulin injections and regular clinic visits concerning glycemic control in diabetic patients, a regression model was employed. Current insulin injections and regular clinic visits were represented as three dummy variables in the model (Fig. 1). Subsequently, the required parameters were input into the Excel table devised by Andersson et al. to calculate the evaluation indexes and 95% confidence intervals (CIs) for additive interactions. The 95% CIs of RERI and AP included 0, while the 95% CI for SI contained 1. These findings indicate the absence of a significant additive interaction between the two influential factors (Table 5).

**Table 5:** Results of additive interaction analysis

Group	Glucose control substandard group (*n* = 155)	Blood glucose control standard group (*n* = 150)	OR95%CI
Currently not required to take insulin injections + not seeing a doctor regularly	52	70	*1*
Currently requires insulin injections + no regular medical visits	4	13	*2.413(0.745 ~ 7.822)*
Currently not required to inject insulin + have regular medical appointments	77	49	*0.473(0.285 ~ 0.785)*
Currently requires insulin injections + has regular medical appointments	13	19	*1.085(0.492 ~ 2.395)*

## Discussion

The management of diabetes mellitus is an ongoing and protracted undertaking. During the initial phases of diabetes mellitus, physicians can identify potential complications for patients with T2DM through physical examination and proactively implement measures to mitigate disease exacerbation. The current study showed that patients with T2DM who received insulin therapy and regular clinic visits had significant effects on their glycemic control. These findings will help improve the management of T2DM patients.

In the present investigation, the results of the multifactorial logistic regression analysis revealed that the OR of insulin therapy was 2.233, indicating a significant association between insulin therapy and glycemic control. This aligns with the findings reported by A S Jarab et al. [7], where the receipt of insulin was associated with an associated with the likelihood of achieving controlled blood glucose. Insulin therapy has emerged as a prevalent and effective strategy for managing fasting blood glucose levels in patients. Studies have demonstrated that individuals without diabetes exhibit pulsatile basal insulin secretion at a rate of 0.5-1.0 U/h. Administering moderate and steady insulin injections after an 8–10 h fasting period has been shown to effectively improve blood glucose levels [8]. Despite these advancements, there remains a subset of patients lacking adequate knowledge regarding preservation. Studies have shown that 67.7% of patients store used insulin in the refrigerator, 2.9% lack understanding of proper preservation methods, and only 29.4% correctly store insulin [9]. Proper storage and administration of insulin play a crucial role in averting disease progression. At the initial visit, patients should be asked whether they have used insulin before. Also, targeted interventions such as regular telephone follow-up by primary care physicians to promote adherence to glucose-lowering medications are import for glucose control of patients with T2DM.

Our results indicate a positive association between regular clinic visits and the achievement of glycemic control. Primary care physicians can set up a schedule of regular visits for patients to test their HbA1c, blood pressure, and so on. Regular visits to the clinic allow patients to have a more in-depth understanding of their condition and obtain prompt expert advice from the primary care physician. A study reported that 72.0% of patients effectively managed their diets, 69.3% adhered to prescribed medications, and only 28.7% maintained regular visits to the outpatient clinics [10]. In recent years, there has been notable progress in the management of hospitals and community healthcare. Numerous organizations are increasingly offering complimentary blood glucose testing for high-risk groups, along with volunteer clinics, publicity, and education. Additionally, scientific literature confirms the effectiveness of chronic disease management through primary care pharmacists [11, 12]. These findings have important implications for healthcare practitioners, highlighting the need to oversee medical services systematically and enhance the overall medical experience for individuals with T2DM.

In contrast to the finding of Hon-Ke Sia [13], the present study demonstrated that exercise habits, dietary practices, smoking history, and alcohol consumption were not statistically significantly associated with blood glucose control. This finding could be explained by the limited awareness of diabetes commonalities among patients, coupled with a lack of standardized education from healthcare professionals. A cross-sectional study of 366 subjects reports that inadequate glucose control and other cardiovascular risk factors are common in the majority of T2DM patients [14]. Among them, 18% of patients have a smoking history, and 14.8% of patients were alcohol users. Medical staff should strengthen patients’ education on smoking and alcohol cessation and instruct patients to exercise regularly. A 12-week study of the Balanced Program underscored the necessity for intensive lifestyle interventions. Timely control of patients’ risk factors by community-based healthcare providers resulted in a 25.7% reduction in the risk of diabetes and cardiovascular disease over a two-year follow-up period [15]. For T2DM patients, lifestyle interventions should be dynamically modified based on varying conditions. Each stage of the disease response presents both similarities and differences, emphasizing the need for tailored lifestyle interventions at different disease stages to achieve optimal blood glucose control [16]. The level of blood glucose affects the condition of diabetic patients. Self-testing of blood glucose serves as an important way for individuals to gain a clear understanding of their condition. Currently, there are intelligent blood glucose testing devices on the market. Patients can connect to the testing devices through their cell phones to monitor their blood glucose levels in real-time and generate test reports, forming the “Internet medical” mode [17]. Studies have concluded that a family history of diabetes, hypertension, and abnormal glucose metabolism is independently associated with the prevalence of abnormal glucose metabolism. The risk of abnormal glucose metabolism increases when these factors coexist. However, no multiplicative or additive interactions were observed between these factors regarding the prevalence of abnormal glucose metabolism [18].

### Strengths and limitations

In this study, we have included a relatively large sample size of subjects to identify factors impacting glycemic control in patients with T2DM. Moreover, this study was conducted in a large tertiary hospital in China, and detailed baseline information has been collected, which is more helpful to fully understand the risk factors that affect the patient’s glycemic control. Lastly, physicians could develop an individualized glucose-lowering plan for patients with T2DM based on these meaningful findings.

The limitations of this study are as follows. Firstly, neither multiplicative nor additive effects between family history and certain independent risk factors reached statistical significance. This observation may be attributed to the fact that the present study was conducted as a single-center retrospective study, rendering it challenging to mitigate potential center-specific effects. Secondly, the relative follow-up period may have led to an underestimation of the impact of certain factors. Further verification through an extended cohort study is warranted to address this limitation comprehensively. Finally, this study was conducted only in a Chinese population. Further research is required to expand the study population to investigate factors associated with glycemic control in patients with T2DM.

## Conclusion

The history of insulin therapy and regular clinic visits were significantly and independently associated with glycated hemoglobin control among patients with T2DM. Improving patients’ proper use of insulin and the habit of regular visits to the doctor largely depends on the interventions implemented by hospitals, communities, and other healthcare organizations. This involves the dissemination and education of diabetes-related knowledge by medical staff to patients. To address the prevailing limited understanding of diabetes among patients and the general public, hospitals and communities should organize diabetes health education activities at reasonable times, focusing on primary prevention. It is crucial to address these aspects to improve overall diabetes management and control.

## Data Availability

The datasets generated and/or analyzed during the current study are available from the corresponding author on reasonable request.

## References

[1] Christ-Crain M, Bichet DG, Fenske WK (2019). Diabetes insipidus. Nat Rev Dis Primers[J].

[2] Zheng Y, Ley SH, Hu FB (2018). Global aetiology and epidemiology of type 2 diabetes mellitus and its complications[J]. Nat Rev Endocrinol.

[3] Al-Qerem W, Jarab AS, Badinjki M (2022). Factors associated with glycemic control among patients with type 2 diabetes: a cross-sectional study[J]. Eur Rev Med Pharmacol Sci.

[4] Habteyohans BD, Hailu BS, Meseret F (2023). Poor glycemic control and its associated factors among children with type 1 diabetes mellitus in Harar, eastern Ethiopia: a cross-sectional study [J]. BMC Endocr Disord.

[5] Zhang N, Du SM, Ma GS (2017). Current lifestyle factors that increase risk of T2DM in China [J]. Eur J Clin Nutr.

[6] [Clinical guidelines for prevention (2022). And treatment of type 2 diabetes mellitus in the elderly in China (2022 edition)]. Zhonghua Nei Ke Za Zhi.

[7] Jarab AS, Al-Qerem W, Alqudah S (2023). Glycemic control and its associated factors in hypertensive patients with type 2 diabetes[J]. Eur Rev Med Pharmacol Sci.

[8] Yang LY. The significance of optimizing insulin dosage for diabetes mellitus glucose management[J]. Chin J Diabetes. 21(12):1146–1149.

[9] Niu LY, Huang J (2013). Problems of insulin use in diabetic patients and the current status of education and management[J]. Chin Nurs J.

[10] Feng JJ, Lan J. A survey study on the knowledge, belief and behavior patterns and health education needs of hospitalized diabetic patients[J]. China Health Educ. 2015(5):508–510.

[11] Benedict AW, Spence MM, Sie JL (2018). Evaluation of a pharmacist-managed diabetes program in a primary care setting within an Integrated Health Care System [J]. J Manag Care Spec Pharm.

[12] Twigg G, Motsko J, Thomas J (2017). Pharmacist-managed diabetes Center interventions Ensure Quality and Safety in Elderly patients [J]. Consult Pharm.

[13] Sla HK, Kor CT, Tu ST (2022). Association between smoking and glycemic control in men with newly diagnosed type 2 diabetes: a retrospective matched cohort study [J]. Ann Med.

[14] Khanal MK, Bhandari P, Dhungana RR (2022). Poor glycemic control, cardiovascular disease risk factors and their clustering among patients with type 2 diabetes mellitus: a cross-sectional study from Nepal [J]. PLoS ONE.

[15] Hu XX, Wei JH, Tian LM (2022). Progress in the application of pre-diabetes interventions and intervention management[J]. Shandong Med.

[16] Chen XB, Jiang MM, GU WJ et al. Construction of evaluation index system for quality control of community diabetes management in different disease stages[J]. China Gen Med. 2020;23(7):837–843.

[17] Wu ZY, Mou X. Progress of Internet+ diabetes management in China[J]. Chin J Diabetes Mellitus. 2022;14(2):204–207.

[18] Xiu ZR (2013). Effect and interaction between family history of diabetes and hypertension on abnormal glucose metabolism[J]. Chin J Diabetes.

